# Excessive Medication

**Published:** 1889-03

**Authors:** 


EXCESSIVE MEDICATION.—We would say to practitioners who read this Journal, don't give so much medicine to your patients. Too much medication is worse than too little for the reason that the *vis medicatrix naturæ* is a powerful ally to the man who practices the expectant plan. The tendency in almost every case is to recovery, and this tendency is often obstructed by harsh remedies and active medication. Watch your patients closely and note the natural efforts of the system to throw off the malady, and encourage the same as best you can. Be cheerful and hopeful in your manner; be patient, sympathetic and kind, let your remedies be few and approprlate to the symptoms, and let the patient get well as he will do in the majority of cases if this plan be pursued.

